# Regeneration Approach to Enhance the Antimicrobial and Antioxidant Activities of Chitosan for Biomedical Applications

**DOI:** 10.3390/polym15010132

**Published:** 2022-12-28

**Authors:** Pradeep Kumar Panda, Kambiz Sadeghi, Kitae Park, Jongchul Seo

**Affiliations:** Department of Packaging & Logistics, Yonsei University, Wonju-si 26493, Gangwon-do, Republic of Korea

**Keywords:** chitosan, regenerated chitosan, polycation, physicochemical properties, antimicrobial activity, antioxidant properties

## Abstract

Owing to its biodegradability, non-toxicity, and biocompatibility, chitosan (Cs) is a ubiquitous biopolymer. However, applications of Cs are limited owing to the existence of strong inter- and intra-molecular hydrogen bonds within its network. To address this issue, we regenerated medium-molecular-weight Cs to enhance the physico-chemical and functional properties using a cationic approach. Accordingly, alkaline modification was employed to introduce an additional positive charge to the amine functional groups of Cs and moderately disintegrate the inter- and intra-hydrogen bonds. The chemical structure of Cs and regenerated chitosan (RCs) was confirmed through Fourier transform infrared and ^1^H-NMR spectroscopy. RCs showed higher zeta potential value compared to Cs. Additionally, using X-ray diffraction, RCs exhibited low crystallinity, which can be attributed to the repulsive force caused by the positive surface charge and the destruction of hydrogen bonds. The RCs exhibited stronger antioxidant activity than Cs. Furthermore, the minimum inhibition concentrations (MICs) of RCs against *Escherichia coli* and *Staphylococcus aureus* were reduced by almost four times compared with those of Cs. The superior functional properties of RCs can be attributed to the formation of a polycationic structure after alkaline modification. Thus, RCs can be introduced as potent agents for various biomedical purposes.

## 1. Introduction

**Figure 1:**
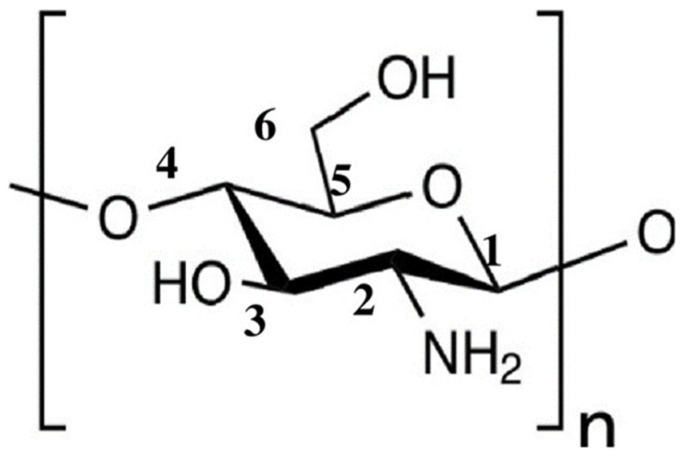
Chemical structure of chitosan.

Chitosan (Cs) is the second most abundant natural linear polysaccharide and multifunctional biopolymer that contains β-(1–4) glucoside bond linkages between d-glucosamine and N-acetyl-d-glucosamine [1,2]. Cs has been utilized for a variety of applications in various industries [3,4,5]. It is derived from chitin via a deacetylation (DD) process [6,7]. Accordingly, depending on the DD and purification processes, Cs can be synthesized with varying molecular weights and degrees of DD. The applications of Cs are significantly dependent upon on its DD and molecular weight (M_w_), which determine its physical characteristics, chemical properties, and functional activities [8,9,10]. Cs is hydrophilic in nature owing to the presence of an amino group at the 2-carbon position, a secondary hydroxyl group at the 3-carbon position, and a primary hydroxyl group at the 6-carbon position [11,12,13] (Figure 1); therefore, Cs is polyatomic in nature. Cs is biodegradable, biocompatible, biologically renewable, nontoxic, non-antigenic, and bio-functional, rendering it attractive for various applications, including drug delivery, tissue engineering, antimicrobial systems, and antibacterial treatments [14,15,16,17,18]. Cs has also been widely used in polymer science as a nanoparticle, especially in the fields of nanomedicine, packaging, and cancer therapy [19,20,21]. Therefore, Cs is a promising natural polymer for application in multidisciplinary fields.

Although Cs has received considerable attention for biological applications, its applicability is still limited owing to the existence of strong inter- and intra-molecular hydrogen bonds within its network and poor solubility in solvents used in the biomedical field, such as water. Recently, numerous studies have focused on improving the water solubility of Cs via chemical modifications [22,23,24]. Nevertheless, chemical modification processes are expensive, time consuming, environmentally unfriendly, and complicated, and present difficulties due to the formation of byproducts [25]. To overcome these challenges, it is necessary to develop a suitable method for improving the functional properties of Cs. For example, the direct addition of natural polysaccharides, such as Cs, into other polymers does not yield a good miscible blend owing to the presence of a strong molecular hydrogen bonding network [26,27]. In this context, Cs can be regenerated via a simple method to improve its physico-chemical properties, including its functional properties. Accordingly, many researchers have tried to synthesize regenerated chitosan using different methods. Generally, an additional hydrogen ion (H^+^) is generated in a Cs solution during the regeneration process, leading to the weakening of the inter- and intra-molecular hydrogen bonds in the Cs macromolecule. Yu et al. fabricated a rubber/regenerated chitin composite system in an aqueous medium. Subsequently, they prepared a composite from the regenerated materials, which demonstrated enhanced functional properties and potential industrial applications [28]. Furthermore, Cs can be regenerated using the ionic liquid method; however, the process is unsafe owing to the formation of byproducts, as well as being expensive and involving complicated purification processes [29]. Regenerated chitosan (RCs) can be applied in many biomedical applications such as drug delivery and tissue engineering. In addition, it has been reported that polycation polymers possess good biocompatibility and bacteriostatic properties, and that, as a result, they could be used as biomaterials [30]. Nonetheless, few studies have focused on increasing the positive charge on the surface of Cs to enhance its biological properties in the quaternized state [31,32,33,34]. However, modifying Cs using this approach is complicated, especially for medium and high molecular Cs. Cs can be modified to its quaternary ammonium salt to enhance its water solubility and, therefore, its functional properties, including antibacterial properties. Ke et al. prepared a quaternary ammonium salt modified Cs microsphere by an emulsion crosslinking reaction for dyeing wastewater treatment [35]. The maintenance of cell viability is important for any application of biomaterials. Hu et al. studied the effect of Cs quaternary ammonium salt on the growth and release of *Microcystis aeruginosa*, showing that it acts as a safe inhibitor [36]. Moreover, some studies have shown that quaternary ammonium salt of Cs has excellent mechanical properties and a homogenous 3D structure [30,37].

To date, only a few studies have focused on regenerating Cs, with special attention dedicated to the improvement of its antioxidant and antimicrobial properties. Therefore, we synthesized regenerated Cs using a simple co-precipitation method with alkaline cationic modification. A positive surface charge was introduced onto the amine functional groups by modifying the inter- and intra-molecular bonds. The chemical structures of Cs and regenerated Cs (RCs) were determined through proton nuclear magnetic resonance and Fourier transform infrared spectroscopy. In addition, the in vitro antibacterial and antioxidant properties were investigated to assess the functional properties of RCs. The crystallinity and thermal stability of the samples were also evaluated.

## 2. Materials and Methods

### 2.1. Materials 

A Cs powder with a medium molecular weight (degree of deacetylation: approximately 85%; M_w_ = 190–310 kDa), agar, deuterated acetic acid (CD_3_COOD), potassium ferricyanide, and deuterated water (D_2_O) were purchased from Sigma-Aldrich (Saint Louis, MO, USA). Sodium hydroxide (NaOH) was also purchased from Sigma-Aldrich (Tokyo, Japan). A nutrient broth was obtained from Becton, Dickinson and Company (Franklin Lakes, NJ, USA). *Escherichia coli* (*E. coli*, DH5α) and *Staphylococcus aureus* (*S. aureus*, ATCC29213) were procured from the Korea Culture Center of Microorganisms (Seoul, South Korea). Phosphate buffer solution (PBS, pH = 7.4), tricholroacetic acid, and ferric chloride were bought from Daejung (Gyeongg-do, South Korea). Furthermore, 2, 2-diphenyl-1-picrylhydrazyl (DPPH) was obtained from Alfa Aesar (Ward Hill, MA, USA). Ethanol, methanol, and acetic acid were obtained from a local vendor in the Republic of South Korea. All reagents and chemicals not listed above were used as received. Deionized (DI) water was used throughout the experiment.

### 2.2. Synthesis of RCs

Cs powder (2 wt%) was dissolved in aqueous acetic acid (1.5 wt%) and stirred at room temperature (23 °C) for 8 h. Subsequently, the sample was completely precipitated using aqueous NaOH (1 wt%). The precipitate was collected using a centrifuge (4500 rpm) for 15 min at room temperature. The sample was rinsed multiple times using a mixture of ethanol and water (1:1 *v*/*v*). Thereafter, the sample was dried in a freeze-dryer (TFD8501, ilShinbiobase Co. Ltd., Dongducheon, South Korea) at −87 °C for 18 h. The dried samples were gently ground using a mortar and pestle and kept in a desiccator cabinet for further analysis.

### 2.3. Characterization

#### 2.3.1. Fourier Transform Infrared (FTIR) Spectroscopy Analysis

The chemical structures of the samples were analyzed using a FTIR (PerkinElmer, Waltham, MA, USA) spectrometer in the attenuated total reflection (ATR) mode. All spectra were collected in the transmission mode (T) within a wavenumber range of 4000–400 cm^−1^ with 32 scans. Air was used as a reference during the analysis.

#### 2.3.2. Proton Nuclear Magnetic Resonance (^1^H-NMR) Analysis 

The chemical structures of the samples were further investigated using a ^1^H-NMR spectrometer (Avance II 400 MHz; Bruker, MA, USA). The samples (5 mg mL^−1^) were initially dissolved in a mixture of CD_3_COOD and D_2_O (1% *v*/*v*). The analysis was performed at ambient temperature, and tetramethylsilane was used as an internal standard. The ^1^H-NMR spectra were recorded, and the chemical shifts were measured in parts per million (ppm).

#### 2.3.3. X-ray Diffraction (XRD) Analysis 

The XRD patterns of the samples were measured using a multipurpose X-ray diffractometer (SmartLab 9 kW system, Rigaku Co., Tokyo, Japan) in a 2θ range of 5–80°. During the analysis, the operating voltage and current were 40 kV and 40 mA, respectively, and the wavelength was 0.154 nm. The degree of crystallinity is the ratio of the sum of the deconvoluted crystalline peak area over the sum of the crystalline and amorphous deconvoluted peak area.

Crystallinity (%) = [A_cr_/(A_cr_ + A_am_)] × 100 (1)

where *A_cr_* and *A_am_* are the integrated area of the crystalline and amorphous peaks, respectively. The integrated areas were calculated using the Origin Software (Origin Lab Co., Northampton, MA, USA).

#### 2.3.4. Analysis of Thermal Properties 

The thermal properties of the samples were determined using thermogravimetric analysis (TGA 4000, PerkinElmer Inc., Waltham, MA, USA) and differential scanning calorimetry (DSC Q-20, TA Instrument Co., DE., New Castle, USA). The thermal stabilities of the samples were investigated using TGA. For this, samples (10–11 mg) were heated from 30 °C to 800 °C at a rate of 10 °C min^−1^. Nitrogen was continuously supplied for purging during the analysis at a rate of 20 mL min^−1^. Graphs were plotted between the weight percentage and temperature (TGA) and derived weight percentage and temperature (DTG). A DSC analysis was conducted as follows: all samples (5–6 mg) were packed into a thermal analysis (TA) aluminum pan and heated from 40 °C to 250 °C at a rate of 10 °C min^−1^ under a continuous flow of nitrogen gas (50 mL min^−1^). Data obtained from the DSC were analyzed using the TA 2000 software.

#### 2.3.5. Zeta Potential Analysis 

The zeta potentials of the Cs and RCs were determined using an ELS-1000ZS Nano Zeta sizer instrument (Otsuka Electronics, Osaka, Japan). The samples were dispersed in DI water at a concentration of 100 ppm. The zeta potentials of the samples were analyzed at room temperature.

#### 2.3.6. Antibacterial Activity Analysis

The antimicrobial activity of the samples was evaluated against *E. coli* (gram-negative) and *S. aureus* (gram-positive) based on the minimum inhibitory concentration (MIC)*,* using the agar plate method. The RCs and Cs solutions (1 wt% *w*/*v*) were prepared using aqueous hydrochloric acid (0.3% *v*/*v*) and autoclaved at 121 °C for 15 min. Samples were prepared with different concentrations (0.1, 0.05, 0.025, 0.0125, 0.00625, 0.00313, and 0.0015625%). Aqueous hydrochloric acid (0.3% *v*/*v*) and zinc oxide (ZnO) were used as the negative and positive controls, respectively. *E. coli* and *S. aureus* suspensions were prepared by transferring a single colony of each microbe into a nutrient broth and tryptic soy broth (10 mL), respectively. The microbial suspensions were incubated at 37 °C for 24 h. Subsequently, an aliquot (1 mL) of each suspension was transferred into each Cs or RCs sample (9 mL) to identify the MIC against bacteria. Sterile distilled water was used to dilute the culture of each bacterium. The number of colonies forming units (CFUs) was calculated per mL; the value was 4.12 × 10^5^. All tests were done at least three times. Bacterial counts were taken by spreading each microbe on an agar plate [38]. 

#### 2.3.7. Antioxidant Activity Analysis 

A DPPH assay was employed to evaluate the antioxidant activity of the samples, with certain modifications [39]. Briefly, DPPH (100 µM) was dissolved in methanol (99.8%). Subsequently, 1 mL of RCs or Cs (1 mg mL^−1^) was incubated with a DPPH solution (1 mL) for 30 min in the dark. The absorbance of the samples was measured using ultraviolet–visible spectroscopy (UV–vis UV-2600i, Shimadzu, Kyoto, Japan) at a wavelength of 517 nm [40,41]. The DPPH scavenging activity (%) was calculated using the following equation:

(2) $$\mathrm{DPPH}\;\mathrm{scavenging}\;\mathrm{activity}\;\left(\%\right)=\left(1-A_{sample}/\;A_{control}\right)\times 100$$

where *A_control_* and *A_sample_* are the absorbance values of the control (without sample) and sample (with DPPH solution), respectively. Each test was performed at least three times.

Ferric reducing power assay was done as per a previous study [2,42]. One milligram of sample was added to a mixture of PBS (2.5 mL) and 1 wt% of aqueous potassium ferricyanide (2.5 mL). The above mixture was heated at 50 °C for 20 min. After cooling to room temperature, 10 wt% of aqueous tricholoroacetic acid (2.5 mL) was added and the mixture was centrifuged at 3000 rpm for 10 min. The upper layer of the mixture (2.5 mL) was mixed with DI water and 0.1 wt% aqueous ferric chloride solution and kept for 10 min. Then, the absorbance at 700 nm was measured. Each experiment was done at least three times.

## 3. Results and Discussion 

### 3.1. FTIR Spectroscopy Analysis

**Figure 2:**
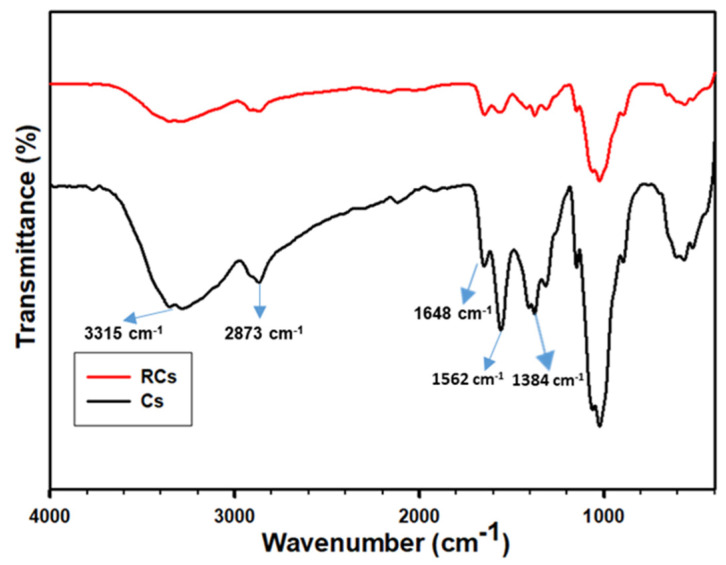
FTIR spectra of Cs and RCs.

A FTIR spectroscopy analysis was conducted to identify the chemical structure of the samples. As illustrated in Figure 2, the Cs and RCs spectra exhibited different peaks related to the chemistry of the samples, as summarized in Table 1. The FTIR spectrum of Cs exhibited several major characteristic bands. The bands at 3570–3000 cm^−1^ correspond to the stretching vibrations of O–H and N–H, and those at 2950–2840 cm^−1^ can be assigned to C–H symmetric and asymmetric stretching [43]. The bands at 1648, 1562, and 1384 cm^−1^ can be attributed to amide I, amide II, and amide III, respectively [44]. Furthermore, C–O stretching and pyranose ring bands were observed at 1064 and 892 cm^−1^, respectively [2]. The peaks corresponding to Cs and RCs were similar, and no new peaks or peak shifts were detected. However, all major bands were slightly shifted to lower wavenumbers. This can be attributed to the disintegration of the inter- and intra-molecular hydrogen bonds in Cs [9]. In addition, chemical changes in the amine functional groups were limited to the surface charges rather than the backbone modifications. The RCs spectrum indicated that the peak intensity was reduced, and the peaks were broadened owing to the destruction of hydrogen bonds within the molecule [1].

### 3.2. ^1^H-NMR Analysis

**Figure 3:**
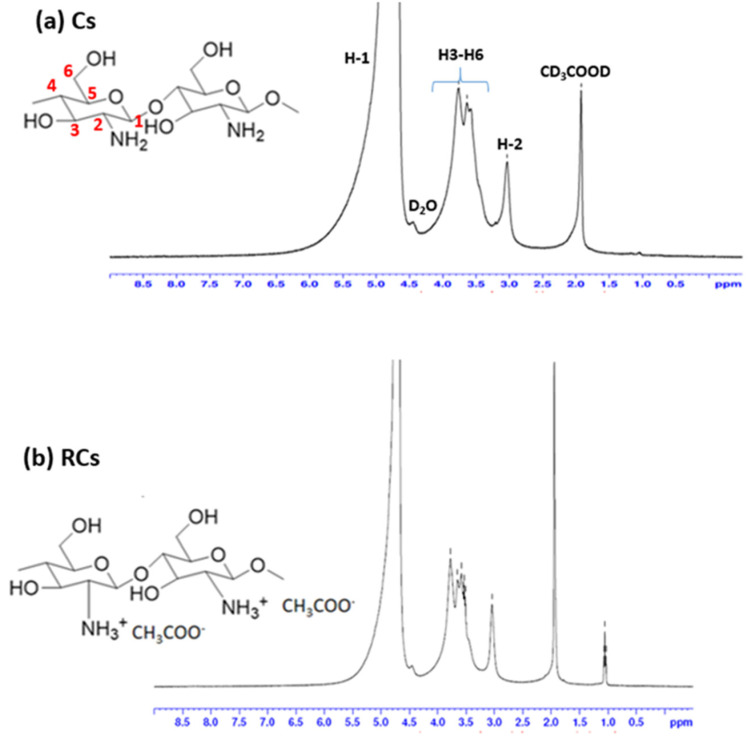
^1^H-NMR spectra of (**a**) Cs and (**b**) RCs.

**Figure 4:**
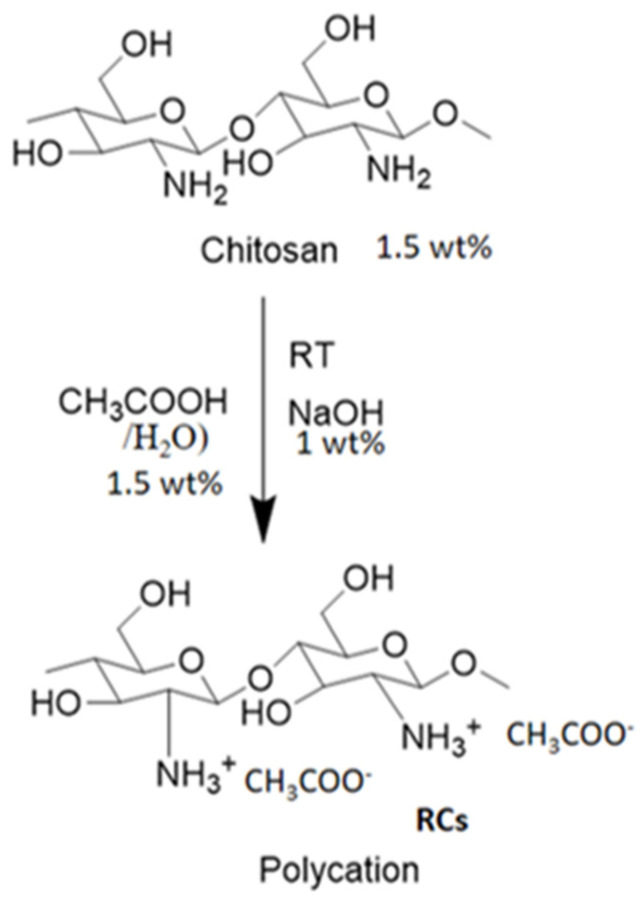
Chemical reaction pathway for synthesis of regenerated chitosan.

The chemical structure was further analyzed using ^1^H-NMR spectroscopy. The ^1^H-NMR spectra of Cs and RCs are depicted in Figure 3. As illustrated in Figure 3a, the chemical shifts at 3.187, 3.710–4.579, and 5.263 ppm can be assigned to H-2, H-3 to H-6, and H-1, respectively, thereby confirming the molecular structure of Cs [1,45]. In addition, the chemical shifts corresponding to RCs were mostly similar to those corresponding to Cs (Figure 3b). A new peak was observed at approximately 1.0 ppm, which might have been due to the residue of the reaction. A possible mechanism behind the chemical reaction is provided in Figure 4. During the chemical reaction, hydrogen ions were formed when the Cs solution was precipitated with NaOH. A hydrogen ion (positive charge) was attached to the amine functional group of the Cs molecule which was difficult to identify using proton NMR spectroscopy. This difficulty might also have been the result of various factors such as the NMR solvent type, reaction conditions, or determination of the exact amount of protonation during the chemical reaction. Moreover, the degree of deacetylation (DD) of the samples was not calculated because the reaction approach was not drastic enough to achieve a deacetylation reaction [46]. It is important to note that no additional peaks were detected in the ^1^H-NMR and FTIR spectra, indicating that the regeneration process employed in this study did not alter the backbone of Cs. The regeneration process was performed to improve the surface charge, which would greatly improve the functional properties of Cs [32,47].

### 3.3. XRD Analysis

**Figure 5:**
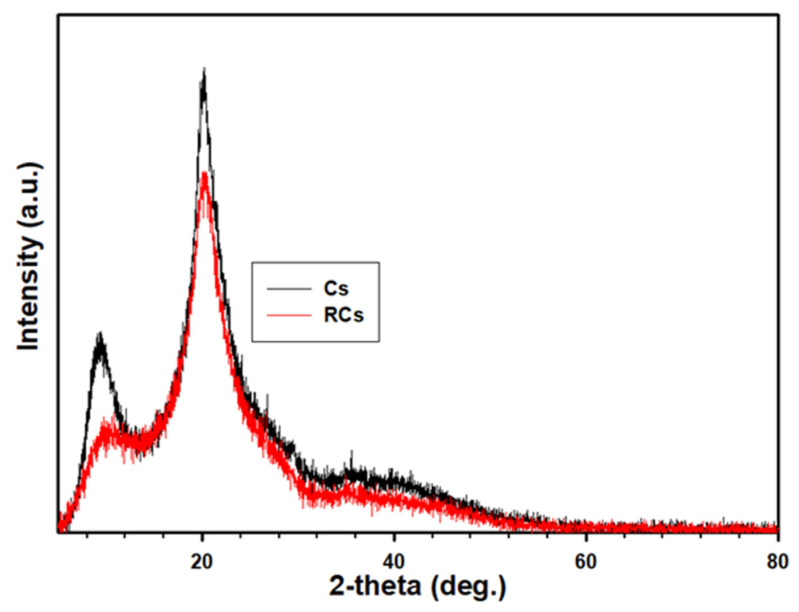
XRD spectra of Cs and RCs.

The XRD pattern of the polymeric sample, depicted in Figure 5, was used to analyze the crystallinity of the sample. The crystallinity of the samples was measured according to the procedure reported in a previous study [48,49], and the results are summarized in Table 2. In both samples, the spectral pattern was similar to the variation in peak intensity (Table 2). Both the samples exhibited main characteristic peaks at approximately 2θ = 20.4°. However, the degree of crystallinity for the samples varied, which could be ascribed to the relative intensity. The relative intensity of Cs was higher than that of RCs, indicating a reduction in the crystallinity. Generally, a polymeric sample exhibiting a sharp peak is considered to be a crystalline material [50]. From this perspective, it can be concluded that the destruction of inter- and intra-molecular bonds tends to reduce the crystallinity of RCs. In addition, the introduction of surface charge might lead to a repulsive force in the materials, interfering with the chain ordering in the crystalline segments of the RCs. Therefore, regeneration in this study led to a reduction in crystallinity, which could enhance the interaction of RCs owing to the higher mobility in the change and functional groups.

### 3.4. Thermogravimetric Analysis (TGA)

**Figure 6:**
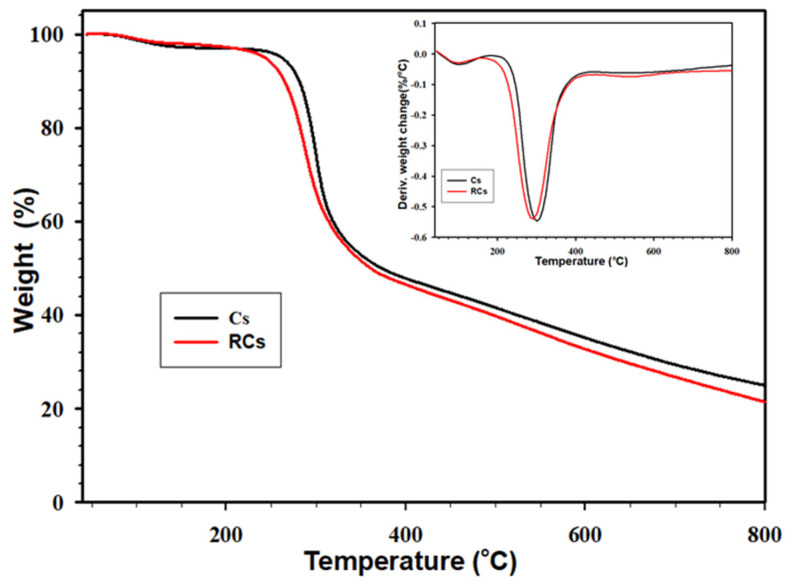
TGA and DTG (Inset) curves of Cs and RCs.

The TGA was employed to determine the thermal stability of the polymeric samples and identify unwanted byproducts produced during regeneration process. The thermal stabilities of the samples are presented in Figure 6. The maximum thermal decomposition temperature was accurately obtained from the DTG curve (Figure 6, inset), and the results are listed in Table 3. As illustrated in Figure 6, both the samples exhibit a similar trend of weight loss at different temperatures. Accordingly, two weight loss stages were found in the samples. The first weight loss stage was found approximately at 50–100 °C owing to the water evaporation. The second weight loss stage at approximately 250–350 °C can be attributed to the decomposition of the Cs backbone. Notably, the second weight loss stage was prominent, which can be observed in the DTG curve. The maximum decomposition temperatures of RCs and Cs were 287 and 302 °C, respectively, confirming the lower thermal stability of RCs than that of Cs. This can be explained by the destruction of inter- and intra-molecular hydrogen bonds in the RCs. In addition, this could be because materials with higher crystallinity are known to exhibit higher thermal stability. Moreover, Table 3 indicates that T_d10%_ (temperature at 10% weight loss) and T_d40%_ (temperature at 40% weight loss) of Cs were significantly higher than those of RCs, which is in agreement with the XRD data. It is worth noting that the decomposition peaks in both the samples were similar, indicating that the regeneration process did not lead to the formation of by-products or unwanted residues, which is consistent with the ^1^H-NMR results.

### 3.5. DSC Analysis 

**Figure 7:**
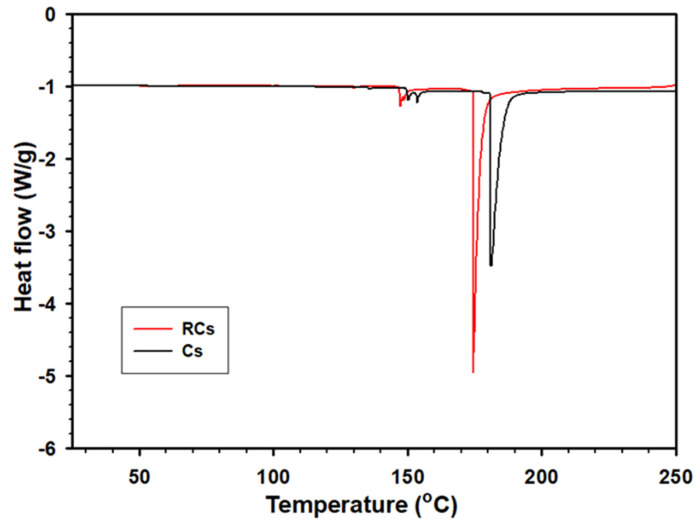
DSC curves of Cs and RCs.

The thermal properties of the samples were investigated via DSC to further confirm the thermal stability of RCs. As depicted in Figure 7, the melting temperature of RCs (174.14 °C) is lower than that of Cs (182.35 °C). Additionally, the melting enthalpies (Δ*H*_m_) of the samples were compared using the results obtained by integrating the area under the melting peak. Accordingly, Δ*H*_m_ of RCs (16.32 J/g) was lower than that of Cs (24.89 J/g), indicating a lower crystallinity. The decrease in the melting point and Δ*H*_m_ of RCs is consistent with their lower crystallinity and change in the molecular bonding, indicating the successful regeneration of Cs. The endothermic peak of RCs was found to be formed at a lower temperature than that of the Cs sample [51].

### 3.6. Zeta Potential Analysis

The exact surface charge of the samples was confirmed using the zeta potential analysis. The zeta potential of Cs was 42.81 mV, whereas that of RCs was 56.61 mV, which is significantly higher. The higher zeta potential of RCs indicates the formation of additional positive surface charges during the chemical reaction. It is known that during the chemical reaction, hydrogen ions dissociate during precipitation and directly attach to the surface of the amine functional group of the Cs molecule, which makes it a polycation. Consequently, the inter- and intra-molecular hydrogen bonds disintegrated completely. Therefore, the functional properties of the RCs were enhanced. 

### 3.7. Antibacterial Activity

The antibacterial activities of the samples, according to the MIC reported in wt%, are presented in Table 4. It was concluded that RCs exhibited higher antimicrobial activities against the microorganisms (*E. coli* and *S. aureus*) used in the study. Additionally, all the samples exhibited a better antimicrobial activity against *E. coli*. ZnO (as a well-known antibacterial agent) [52] and HCl were used as the positive and negative controls, respectively. Notably, the synthesized RCs exhibited a higher antibacterial activity against both microorganisms compared with ZnO.

Generally, polycationic materials exhibit better antibacterial activities than corresponding virgin polyatomic materials [53]. Cs is a macromolecule that can’t easily pass through the outer membrane of bacteria [54]. Therefore, a direct access to Cs to the intracellular parts of the cell is unlikely. Accordingly, the antibacterial mechanisms of Cs have been suggested. The positive charge of the amino group at C-2 in the polycationic structure can interact with the anionic components (lipopolysaccharides and proteins) present predominantly on the surface of the microorganism. The interaction results in an intense deformation at the outer membrane structure, resulting in a leakage of proteinaceous cells [37]. The regeneration of Cs into RCs introduces additional positive charges on the amino groups of the RCs. Consequently, RCs further interacts with the anionic components of the cell surface and inhibits bacterial growth at a lower concentration. The antibacterial potency of RCs against *E. coli* was different from that for *S. aureus* owing to differences in the cell wall structure. Gram-positive bacteria (e.g., *S. aureus*) possess higher peptidoglycans (thicker cell walls) than gram-negative bacteria (e.g., *E. coli*). Therefore, RCs exhibited stronger antimicrobial properties than Cs [55]. Apart from the above reasons, antibacterial activity of Cs highly depends upon its molecular weight, concentration, and number of moles of the amino group [56]. 

### 3.8. Antioxidant Activity

The DPPH and reducing power assay were used to evaluate the antioxidant activity of the samples. DPPH is a stable free radical that accepts an electron or hydrogen atom. The radical scavenging activity (%) of the samples was evaluated by measuring the absorbance of the samples at a wavelength of 517 nm. The results are presented in Table 5. Gallic acid was used as the positive control, as it is a well-known antioxidant. The radical scavenging activities (%) of Cs, RCs, and gallic acid were 45.26, 87.32, and 95.44%, respectively. The RCs demonstrated potent antioxidant activity, comparable to that of the positive control (Gallic acid) in the same condition. Additionally, the RCs exhibited a significant increase in the radical scavenging activity (87.32%), indicating higher antioxidant effects. The radical scavenging activity of Cs might be related to the reaction of free radicals with the hydroxyl group at the C-6 position through H-abstraction and the reaction of free radicals with the amino group at the C-2 position to form a stable macromolecule [2,57]. Cs is known to possess a compact structure owing to the presence of stronger inter- and intra-molecular hydrogen bonds inside the macromolecule. Therefore, it exhibits a high viscosity and low mobility for interactions with radical species. Conversely, RCs, with a higher positive charge (additional hydrogen), lower inter- and intra-molecular hydrogen bonding, low viscosity, and higher mobility, tends to readily donate a hydrogen atom to each DPPH radical molecule and stabilize the radical species. Therefore, RCs was found to exhibit higher antioxidant activities, enabling its use as a strong antioxidant.

A reducing power assay was performed; the data are shown in Table 5. In this assay, Fe^3+^ is converted to Fe^2+^, which reacts with ferric chloride solution, forming a ferrous complex. The maximum absorption value of ferrous complex is 700 nm. The more absorbance of the reaction mixture showed more reducing power. In Table 5, Cs has an absorption of 0.36, whereas RCs has a value of 88.21, which is significantly higher. RCs donates electrons more readily than Cs due to its enhanced reducing power, which accelerates the formation of Fe^2+^. The above results show that RCs has more antioxidant activity than Cs, comparable with the positive control (Gallic acid). This result is in agreement with the DPPH assay.

## 4. Conclusions

In this study, medium molecular weight Cs was regenerated to RCs with a polycationic structure using a facile alkaline cationic treatment process. FTIR spectra and ^1^H-NMR spectroscopy confirmed that the backbone of Cs had remained unaltered, indicating that the regeneration was restricted to the functional groups present on the amine group. In addition, a decrease in the melting point, Δ*H*_m_, (34.43% decrease) and thermal decomposition confirmed the lower crystallinity of RCs owing to the introduction of a positive surface (repulsive force) and the destruction of hydrogen bonds. Alkaline regeneration resulted in the formation of a polycationic structure on the amine functional groups of RCs, strengthening the interaction of this material with radical species and bacteria. The simple cationic regeneration process yielded enhanced antimicrobial properties against representative bacteria (MICs of RCs were reduced almost four-fold more compared to those of Cs) and a higher antioxidant activity (42.06% enhanced in DPPH assay and 52% enhanced in FRAP assay). The results for functional properties tests were comparable with those of the positive controls, indicating the strong functionality of RCs. The modification of Cs using NaOH might be varied depending on the NaOH concentration. As a result, a lower or higher concentration of NaOH might lead to Cs with different properties, although this needs to be studied. Therefore, the RCs prepared using this new facile strategy could be used as an antimicrobial and antioxidant compound in biomedical applications.

## Figures and Tables

**Table 1:** FTIR bands of Cs and RCs with peak assignments.

Sample	Peak Assignments (cm^−1^)	Reference
Cs	O-H and N-H stretching (3315); C–H symmetric and asymmetric stretching (2873); Amide I (1648); Amide II (1562); Amide III (1384)	[9,43,44]
RCs	O-H and N-H stretching (3292); C–H symmetric and asymmetric stretching (2865); Amide I (1639); Amide II (1547); Amide III (1372)	[1,9,44]

**Table 2:** Representative values of XRD.

Sample	Intensity (a. u.)	Relative Intensity (%)	Crystallinity (%)
Cs	3144 ± 5	100.0 ± 0.00	63.02 ± 0.8
RCs	2444 ± 3	77.73 ± 0.03	49.52 ± 1.7

**Table 3:** Representative thermal properties (TGA and DTG) values of Cs and RCs.

Sample	T_d10%_ (°C)	T_d40%_ (°C)	Residue (%)	DTG (°C)
Cs	281 ± 1	316 ± 1	25.02 ± 0.51	302 ± 2
RCs	264 ± 2	311 ± 1	21.12 ± 0.64	287 ± 3

**Table 4:** Antibacterial activity of Cs and RCs.

Strain	Cs (% *w*/*v*)	RCs (% *w*/*v*)	Positive Control (ZnO % *w*/*v*)	Negative Control (HCl % *w*/*v*)
*E. coli*	0.01250	0.00313	0.025	0.05
*S. aureus*	0.02500	0.00625	0.050	0.10

**Table 5:** Antioxidant activity of the Cs and RCs.

Sample	DPPH Radical Scavenging Activity (%)	Absorbance at 700 nm
Cs RCs Gallic acid	45.26 ± 1.20 87.32 ± 0.43 95.44 ± 0.98	0.36 ± 0.08 0.88 ± 0.12 0.93 ± 0.11

## Data Availability

Not applicable.

## References

[1] Panda P.K., Yang J.M., Chang Y.H. (2021). Water-induced shape memory behavior of poly (vinyl alcohol) and p-coumaric acid-modified water-soluble chitosan blended membrane. Carbohydr. Polym..

[2] Panda P.K., Yang J.M., Chang Y.H., Su W.W. (2019). Modification of different molecular weights of chitosan by p-Coumaric acid: Preparation, characterization and effect of molecular weight on its water solubility and antioxidant property. Int. J. Biol. Macromol..

[3] Aider M. (2010). Chitosan application for active bio-based films production and potential in the food industry. LWT-Food Sci. Techonol..

[4] Pokharel S., Yadav P.N., Adhikari R. (2015). Applications of chitin and chitosan in industry and medical science: A review. Nepal. J. Sci. Technol..

[5] Song Z., Li G., Guan F., Liu W. (2018). Application of chitin/chitosan and their derivatives in the papermaking industry. Polymer.

[6] Honary S., Ghajar K., Khazaeli P., Shalchian P. (2011). Preparation, characterization and antibacterial properties of silver-chitosan nanocomposites using different molecular weight grades of chitosan. Trop J. Pharm. Res..

[7] Kumar M.N.R. (2000). A review of chitin and chitosan applications. React. Funct. Polym..

[8] Bof M.J., Bordagaray V.C., Locaso D.E., García M.A. (2015). Chitosan molecular weight effect on starch-composite film properties. Food Hydrocoll..

[9] Panda P.K., Dash P., Chang Y.H., Yang J.M. (2022). Improvement of chitosan water solubility by fumaric acid modification. Mater. Lett..

[10] Liu Z., Ge X., Lu Y., Dong S., Zhao Y., Zeng M. (2012). Effects of chitosan molecular weight and degree of deacetylation on the properties of gelatine-based films. Food Hydrocoll..

[11] Luan F., Wei L., Zhang J., Mi Y., Dong F., Li Q., Guo Z. (2018). Antioxidant activity and antifungal activity of chitosan derivatives with propane sulfonate groups. Polymers.

[12] Panda P.K., Yang J.M., Chang Y.H. (2021). Preparation and characterization of ferulic acid-modified water-soluble chitosan and poly (gamma-glutamic acid) polyelectrolyte films through layer-by-layer assembly towards protein adsorption. Int. J. Biol. Macromol..

[13] Yang J.M., Panda P.K., Jie C.J., Dash P., Chang Y.H. (2022). Poly (vinyl alcohol)/chitosan/sodium alginate composite blended membrane: Preparation, characterization, and water-induced shape memory phenomenon. Polym. Eng. Sci..

[14] Mi F.L., Tan Y.C., Liang H.F., Sung H.W. (2022). In vivo bicompatibility and degradability of a novel injectable-chitosan-based implant. Biomaterials.

[15] Panda P.K., Dash P., Yang J.M., Chang Y.H. (2022). Development of chitosan, graphene oxide, and cerium oxide composite blended films: Structural, physical, and functional properties. Cellulose.

[16] Tamer T.M., Valachová K., Hassan M.A., Omer A.M., El-Shafeey M., EldinŠoltés M.S.M. (2018). Chitosan/hyaluronan/edaravone membranes for anti-inflammatory wound dressing: In vitro and in vivo evaluation studies. Mater. Sci. Eng. C.

[17] Tamer T.M., Collins M.N., Valachová K., Hassan M.A., Omer A.M., Mohy-Eldin M.S., Švík K., Jurčík R., Ondruška Ľ., Biró C. (2018). MitoQ loaded chitosan-hyaluronan composite membranes for wound healing. Materials.

[18] Yildirim-Aksoy M., Beck B. (2017). Antimicrobial activity of chitosan and a chitosan oligomer against bacterial pathogens of warmwater fish. J. Appl. Microbiol..

[19] Ahmad M.Z., Rizwanullah M., Ahmad J., Alasmary M.Y., Akhter M.H., Abdel-Wahab B.A., Warsi M.H., Haque A. (2022). Progress in nanomedicine-based drug delivery in designing of chitosan nanoparticles for cancer therapy. Int. J. Polym. Mater. Polym. Biomater..

[20] Kim D.S., Dhand V., Rhee K.Y., Park S.J. (2015). Study on the effect of silanization and improvement in the tensile behavior of graphene-chitosan-composite. Polymers.

[21] Azizian S., Hadjizadeh A., Niknejad H. (2022). Chitosan-gelatin porous scaffold incorporated with Chitosan nanoparticles for growth factor delivery in tissue engineering. Carbohydr. Polym..

[22] Gomez-Maldonado D., Filpponen I., Hernandez-Diaz J.A., Waters M.N., Auad M.L., Johansson L.S., Peresin M.S. (2021). Simple functionalization of cellulose beads with pre-propargylated chitosan for clickable scaffold substrates. Cellulose.

[23] Huaytragul J., Chalitangkoon J., Monvisade P., Chotsaeng N. (2021). Enhancing chitosan solubility in alcohol: Water mixtures for film-forming systems releasing with turmeric extracts. J. Taiwan Inst. Chem. Eng..

[24] Kandile N.G., Mohamed M.I., Zaky H.T., Nasr A.S., Ali A.G. (2022). Quinoline anhydride derivatives cross-linked chitosan hydrogels for potential use in biomedical and metal ions adsorption. Polym. Bull..

[25] Abdelhamid H.N. (2022). Chitosan-Based Nancarriers for Gene Delivery. Nanoeng. Biomater..

[26] Huang B., He H., Liu H., Zhang Y., Chen H., Ma Y. (2020). Co-precipitated poly (vinyl alcohol)/chitosan composites with excellent mechanical properties and tunable water-induced shape memory. Carbohydr. Polym..

[27] Huang B., He H., Liu H., Wu W., Ma Y., Zhao Z. (2019). Mechanically strong, heat-resistant, water-induced shape memory poly (vinyl alcohol)/regenerated cellulose biocomposites via a facile co-precipitation method. Biomacromolecules.

[28] Yu P., He H., Luo Y., Jia D., Dufresne A. (2017). Elastomer reinforced with regenerated chitin from alkaline/urea aqueous system. ACS Appl. Mater. Interfaces.

[29] Sun X., Huang C., Xue Z., Mu T. (2015). An environmentally benign cycle to regenerate chitosan and capture carbon dioxide by ionic liquids. Energy Fuels.

[30] Geng Z., Ji Y., Yu S., Liu Q., Zhou Z., Guo C., Pei D. (2021). Preparation and characterization of a dual cross-linking injectable hydrogel based on sodium alginate and chitosan quaternary ammonium salt. Carbohydr. Res..

[31] Guo Z., Xing R., Liu S., Zhong Z., Ji X., Wang L., Li P. (2007). Antifungal properties of Schiff bases of chitosan, N-substituted chitosan and quaternized chitosan. Carbohydr. Res..

[32] Martins A.F., Facchi S.P., Follmann H.D., Pereira A.G., Rubira A.F., Muniz E.C. (2014). Antimicrobial activity of chitosan derivatives containing N-quaternized moieties in its backbone: A review. Int. J. Mol. Sci..

[33] Pasquina-Lemonche L., Burns J., Turner R.D., Kumar S., Tank R., Mullin N., Hobbs J.K. (2020). The architecture of the Gram-positive bacterial cell wall. Nature.

[34] Wang C.H., Liu W.S., Sun J.F., Hou G.G., Chen Q., Cong W., Zhao F. (2016). Non-toxic O-quaternized chitosan materials with better water solubility and antimicrobial function. Int. J. Biol. Macromol..

[35] Ke P., Zeng D., Xu K., Cui J., Li X., Wang G. (2020). Preparation of quaternary ammonium salt-modified chitosan microspheres and their application in dyeing wastewater treatment. ACS Omega.

[36] Zhu Y., Pei H., Hu W., Jin Y., Xu H., Ren Y., Xue D. (2016). Effect of chitosan quaternary ammonium salt on the growth and microcystins release of Microcystis aeruginosa. RSC Adv..

[37] Jiang S., Wang L., Yu H., Chen Y. (2005). Preparation of crosslinked polystyrenes with quaternary ammonium and their antibacterial behavior. React. Funct. Polym..

[38] Speciale A., Musumeci R., Blandino G., Milazzo I., Caccamo F., Nicoletti G. (2002). Minimal inhibitory concentrations and time-kill determination of moxifloxacin against aerobic and anaerobic isolates. Int. J. Antimicrob. Agents.

[39] Woranuch S., Yoksan R. (2013). Preparation, characterization and antioxidant property of water-soluble ferulic acid grafted chitosan. Carbohy Polym..

[40] Kim H., Panda P.K., Sadeghi K., Lee S., Chung C., Park Y., Seo J. (2022). Facile thermal and hydrolytic conversion of tannic acid: Enhancement of antimicrobial activity and biocompatibility for biomedical applications. Mater. Chem. Phys..

[41] Kim H., Panda P.K., Sadeghi K., Seo J. (2023). Poly (vinyl alcohol)/hydrothermally treated tannic acid composite films as sustainable antioxidant and barrier packaging materials. Prog. Org. Coat..

[42] Oyaizu M. (1986). Studies on products of browning reaction antioxidative activities of products of browning reaction prepared from glucosamine. Japan. J. Nutri. Dietei..

[43] Liu J., Lu J.F., Kan J., Tang Y.Q., Jin C.H. (2013). Preparation, characterization and antioxidant activity of phenolic acids grafted carboxymethyl chitosan. Int. J. Biol. Macromol..

[44] Zhang C., Ping Q., Zhang H., Shen J. (2003). Synthesis and characterization of water-soluble O-succinyl-chitosan. Euro. Polym. J..

[45] Wiessler M., Waldeck W., Kliem C., Pipkorn R., Braun K. (2010). The Diels-Alder-reaction with inverse-electron-demand, a very efficient versatile click-reaction concept for proper ligation of variable molecular partners. Int. J. Med. Sci..

[46] Hsu C.H., Chen S.K., Chen W.Y., Tsai M.L., Chen R.H. (2015). Effect of the characters of chitosans used and regeneration conditions on the yield and physicochemical characteristics of regenerated products. Int. J. Mol. Sci..

[47] Li C., Han Q., Guan Y., Zhang Y. (2014). Thermal gelation of chitosan in an aqueous alkali–urea solution. Soft Matter..

[48] Ioelovich M. (2014). Crystallinity and hydrophility of chitin and chitosan. J. Chem..

[49] Park K., Sadeghi K., Panda P.K., Seo J., Seo J. (2022). Ethylene vinyl acetate/low-density polyethylene/oyster shell powder composite films: Preparation, characterization, and antimicrobial properties for biomedical applications. J. Taiwan Inst. Chem. Eng..

[50] Panda P.K., Dash P., Biswal A.K., Chang Y.H., Misra P.K., Yang J.M. (2022). Synthesis and Characterization of Modified Poly (vinyl alcohol) Membrane and Study of Its Enhanced Water-Induced Shape-Memory Behavior. J. Polym. Environ..

[51] Zeng L., Qin C., Wang L., Li W. (2011). Volatile compounds formed from the pyrolysis of chitosan. Carbohy. Polym..

[52] Barreto M.S., Andrade C.T., da Silva L.C.R., Cabral L.M., Flosi Paschoalin V.M., Del Aguila E.M. (2017). In vitro physiological and antibacterial characterization of ZnO nanoparticle composites in simulated porcine gastric and enteric fluids. BMC Vet. Res..

[53] Tan H., Ma R., Lin C., Liu Z., Tang T. (2013). Quaternized chitosan as an antimicrobial agent: Antimicrobial activity, mechanism of action and biomedical applications in orthopedics. Int. J. Mol. Sci..

[54] Helander I.M., Latva-Kala K., Lounatmaa K. (1998). Permeabilizing action of polyethyleneimine on Salmonella typhimurium involves disruption of the outer membrane and interactions with lipopolysaccharide. Microbiology.

[55] Gan L., Chen S., Jensen G.J. (2008). Molecular organization of Gram-negative peptidoglycan. Proc. Natl. Acad. Sci. USA.

[56] Ortega-Ortiz H., Gutiérrez-Rodríguez B., Cadenas-Pliego G., Jimenez L. (2010). Antibacterial activity of chitosan and the interpolyelectrolyte complexes of poly (acrylic acid)-chitosan. Braz. Arch. Biol. Technol..

[57] Xie W., Xu P., Liu Q. (2001). Antioxidant activity of water-soluble chitosan derivatives. Bioorg. Med. Chem. Lett..

